# Secrecy outage probability and strictly positive secrecy capacity of UAV assisted cooperative NOMA system with two untrusted destinations

**DOI:** 10.1038/s41598-025-92321-0

**Published:** 2025-03-10

**Authors:** Mohd Javed Khan, Saif Ahmad, Indrasen Singh, Umesh Kumar Sahu

**Affiliations:** 1 Department of Electronics and Communication Engineering, Integral University, Lucknow, UP India; 2 Department of Embedded Technology, School of Electronics Engineering, Vellore Institute of Technology, Vellore, Tamil Nadu 632014 India; 3 Department of Mechatronics, Manipal Institute of Technology, Manipal Academy of Higher Education, Manipal, Karnataka 576104 India

**Keywords:** Non-orthogonal multiple access (NOMA), Unmanned aerial vehicle (UAV), Amplify-and-forward relay (AFR), Secrecy outage probability (SOP), Strictly positive secrecy capacity (SPSC), Aerospace engineering, Engineering

## Abstract

The volume of confidential information transmitted over 5G networks has increased rapidly due to the widespread adoption of large machine-type communication and Internet of Things (IoT) devices. Secrecy outage probability (SOP) and strictly positive secrecy capacity (SPSC) parameters are crucial parameters used in evaluating the security of wireless systems, particularly in situations where maintaining secrecy is essential. Also, Non-orthogonal multiple access (NOMA) has the potential to improve the performance of wireless communication systems due to its higher spectral efficiency, improved fairness in resource allocation, and enhanced coverage and connectivity. In this paper, we investigate the secrecy performance of unmanned aerial vehicles (UAV) assisted cooperative NOMA system with a half duplex protocol based amplify and forward relay over the Rayleigh fading channel in presence of two eavesdroppers for downlink communication. We derive SOP and SPSC of the proposed system model and analyze its performance in terms of the illegal SNR of eavesdroppers, power allocation coefficient, information rates and Rayleigh channel parameters. Simulation results demonstrate that SOP decreases with a higher value of power allocation coefficient and lower values of the illegal SNR of eavesdropper and lower information rates. SPSC increases with high values of power allocation coefficient of far destination and decreases with lower values of illegal SNR of eavesdroppers and Rayleigh channel parameters. Analytical simulations are verified using Monte Carlo simulations.

## Introduction

The aims of sixth generation (6G) network is to provide the ultra-high spectral efficiency, massive device connectivity ultra-reliable low-latency communications (URLLC) and superior energy efficiency^1^ . It is expected to support emerging technologies like the Internet of Things (IoT) and applications like augmented reality (AR) and virtual reality (VR)^2^. Although the number of cutting-edge IoT devices has increased and network performance has improved, personal IoT devices have also brought up critical privacy and security issues^3,4^. In actuality, there is a significant chance of eavesdropping during content transfer in wireless networks because of the broadcast feature of wireless signals. To solve these problems, reliable physical layer security methods are needed that protect from both internal and external attacks and maintain data confidentiality and integrity in real-time situations. These issue of security can be solved using physical layer security^5^.

Non- Orthogonal Multiple Access (NOMA) has gained substantial attention in recent times as it allows multiple users to share the same time and frequency resources, increasing spectral efficiency and user capacity. On the other hand, Orthogonal Multiple Access (OMA) allocates separate resources to each user, limiting the number of simultaneous connections. NOMA offers several advantages over OMA that make it an appealing choice for wireless communication systems. Firstly, NOMA significantly enhances spectral efficiency by allowing multiple users to share the same resources simultaneously. This is achieved through power domain multiplexing, where users with better channel conditions are allocated more power than those with weaker channels. As a result, NOMA can support a larger number of users within the same bandwidth compared to OMA, making it an efficient solution for overcrowded networks. The NOMA approach has the potential to be integrated with current multiple access paradigms since it takes advantage of the new aspect of the power domain^6^. In Ref.^7^, power domain NOMA was discussed in detail and also compared with code domain NOMA. Furthermore, a comparative analysis of the various NOMA techniques’ methodologies concerning the receiver’s complexity as a result of successive interference cancellation (SIC) was also covered. Cooperative NOMA was used in Ref.^8^ to enhance the performance of far-user with poor channel condition. Cooperative transmission can construct a virtual multiple-input multiple-output (MIMO) system to process data collaboratively, which can improve communication dependability for users under weak channel circumstances.

Unmanned aerial vehicles (UAVs) that can be utilized for a variety of purposes such as military, surveillance tasks, farming and agricultural systems delivery of goods and healthcare services, aerial photography, and surveying are also made feasible by 6G^9,10^. 6G is a technology suitable for civilian as well as military UAV networks. It can serve as an essential technology in communication networks^11–14^. Recently, several works have been done to improve the performance of UAVs. In Ref.^15^, a task scheduling algorithm was proposed for Space-Air-Ground integrated networks to ensure fairness in resource allocation. In Ref.^16^, a path loss and shadowing model was developed for UAV-ground communication, considering urban infrastructure impact. Deep reinforcement learning (DRL) and Boids based model were analyzed for UAV pursuit-evasion methods in Ref.^17^. A causal intervention approach for target-driven navigation was analyzed in Ref.^18^. Wavelet decomposition was used in Ref.^19^ to present a deep learning-based anomaly detection technique for UAV networks. A CSMA/CA-based optimization methodology was presented in Ref.^20^ to strike a compromise between efficiency and fairness in UAV-MIMO networks. Age of information limitations in ultra-reliable low-latency communications (URLLC) UAV networks were examined in Ref.^21^. In Ref.^22^, an energy consumption model was analyzed for UAV assisted communications. In Ref.^23^,an adaptive control system for UAV navigation using DRL was analyzed. In Ref.^24,25^, different parameters of UAV systems were analyzed for enhancing accuracy. In Ref.^26^, a switching control strategy for multiple UAVs was developed for omni-directional intelligent navigator.

Additionally, UAVs can increase coverage area and assist small base stations in offloading traffic^27^. Therefore, it is important to provide the security on the physical layer of UAV networks^28^. With NOMA, UAVs can transmit and receive data simultaneously^29^, utilizing the power domain for user separation. This allows for a higher number of UAVs to operate in the same area without causing interference or congestion. Additionally, NOMA’s low-latency capabilities make it suitable for real-time applications, such as UAV control and video streaming. Unfavorable weather, such as rain, snow, or strong winds, might interfere with all UAV-assisted communication. Signal quality for UAV-assisted communication might fluctuate suddenly due to fading, interference, and other issues. Since all users in wireless networks receive messages over an open channel, users must protect their message confidentiality from both external and internal eavesdroppers. One interesting approach to improving the secrecy performance of system is to take advantage of physical layer capabilities of wireless networks.

Cooperative transmission applied to NOMA has recently been taken into consideration in Ref.^8,30,31^. In Ref.^30^, a cooperative NOMA system with several relays was examined. A hybrid decode-and-forward (DF) and amplify-and-forward (AF) relaying method were used in Ref.^30^. The AF relay first amplifies the received signal before forwarding it to the destination, while the DF relay first decodes the received signal and then forwards it to the destination after re-encoding. The performance of a NOMA-based system using DF relaying of a two-stage relay selection (TSRS) approach during outages was examined in Ref.^32^. According to the results in Ref.^32^, the suggested TSRS performs better than the typical max–min relay selection method^33^. More recently, the authors in Ref.^34^ investigated a NOMA based cooperative system using an AF relay and came up with a reasonable approximation for the outage probability. Wyner was the first to put up the idea of physical layer security due to the wireless communication broadcast nature^35^.

Physical layer security has lately attracted a lot of interest as an interesting way to create secure communication. A lot of research has been done on secrecy performance analysis for many systems up to this point, including MIMO (transmit antenna selection and cooperative diversity^36^, maximum ratio combining)^37^, cognitive radio^38^ and energy harvesting^39^. These works, however, have no relation to the cooperative NOMA system. The physical layer security for 5G NOMA has recently been taken into consideration in^40,41^ using stochastic geometry. Later, a continuation of^40^ was made and reported in^42^, where two new structures were suggested to enhance the secrecy performance for single and multiple antenna probability based stochastic geometrical systems, respectively. The best designs of power, transmission rates, and decoding order given to each user were explored in^43–45^, along with a novel NOMA design under secrecy concerns. NOMA has been seen as a potentially useful approach for downlink communication because of its enhanced spectral efficiency and its capacity to provide large-scale connectivity^46–49^. The study conducted in^50^ examines the secrecy outage probability (SOP) of a NOMA system that has external eavesdroppers. The study in Ref.^51^ used maximum ratio transmission (MRT) and maximum ratio combination (MRC) techniques to investigate the SOP of MIMO-NOMA relay systems. In the same way, the effect of imperfect channel state information (CSI) on SOP for cooperative cognitive NOMA networks is investigated in Ref.^52^. The secrecy performance of downlink overlay CR-NOMA systems with imperfect SIC was examined in^53^. The SOP of the other NOMA systems that are susceptible to external eavesdropping have been examined in a variety of contexts, including secure cooperative NOMA systems and relay-assisted NOMA networks^54^. The study in Ref.^55^ used long short-term memory-based channel estimation to improve security and performance while examining energy efficiency maximization in device-to-device-based cognitive radio networks using NOMA while taking eavesdropping concerns into account. Likewise,^56^ examined an end-to-end cooperative NOMA-based Internet of Things system with wireless energy harvesting, assessing the likelihood of a secrecy outage and determining the best power distribution to enhance secrecy. UAV assisted cooperative NOMA systems were studied recently to analyze the systems performance^57–59^. The study conducted in Ref.^50^ examines the secrecy outage probability (SOP) of a NOMA system that has external eavesdroppers. The SOP of the other NOMA systems that are susceptible to external eavesdropping have been examined in a variety of contexts, including secure cooperative NOMA systems and relay-assisted NOMA networks^54^. UAV assisted cooperative NOMA systems were studied recently to analyze the systems performance^57–59^

### Motivation and contribution

With the evaluation of 6G networks, the demand for high spectral efficiency and massive connectivity is increasing rapidly. In this context, UAV is a promising technique to enhance the network performance. UAVs are also useful in distant and disaster-affected areas because they provide adaptable and increased coverage. Simultaneously, NOMA has also gained attention due to its higher spectral efficiency and huge connectivity in comparison with the orthogonal multiple access technique. Recent research also shows that UAVs can maximize user access and network throughput when used with NOMA. However, UAV-assisted communications are susceptible to eavesdropping and security threats due to the coexistence of trusted and untrusted users in the open nature of wireless communication. Physical layer security (PLS) is a key technique to enhance the secrecy performance of wireless networks without depending on conventional cryptographic techniques. Although the PLS of NOMA systems has been investigated thoroughly, a secrecy performance of UAV-assisted cooperative NOMA systems in the presence of untrusted destinations have not yet been analyzed. The impact of power allocation coefficient, illegal SNR of eavesdroppers, and Rayleigh fading parameters on secrecy metrics such as SOP and Strictly Positive Secrecy Capacity (SPSC) has not been investigated thoroughly yet.

The major contributions and novelty of this work are as follows:

- We propose a UAV assisted cooperative NOMA system with two untrusted destinations which uses a half-duplex (HD) AF relay over Rayleigh fading channel for downlink communication.
- We derive the analytical expressions of SOP, SPSC, and asymptotic SOP and analyze the secrecy performance in terms of average values of illegal SNR, target rate, power allocation coefficient, and Rayleigh channel parameters.
- We use Monte Carlo simulations to validate the analytical results.

### Organization of the paper

The remaining content of this paper is written as follows: In the second section, we propose and describe the UAV assisted cooperative NOMA system with two untrusted destinations which uses AF relay. In the third section, we derive the SOP and SPSC of proposed system with HD AF relay. In the fourth section, we show the outcomes of the simulation results and in the fifth section, we conclude the paper.

## System model

**Figure 1:**
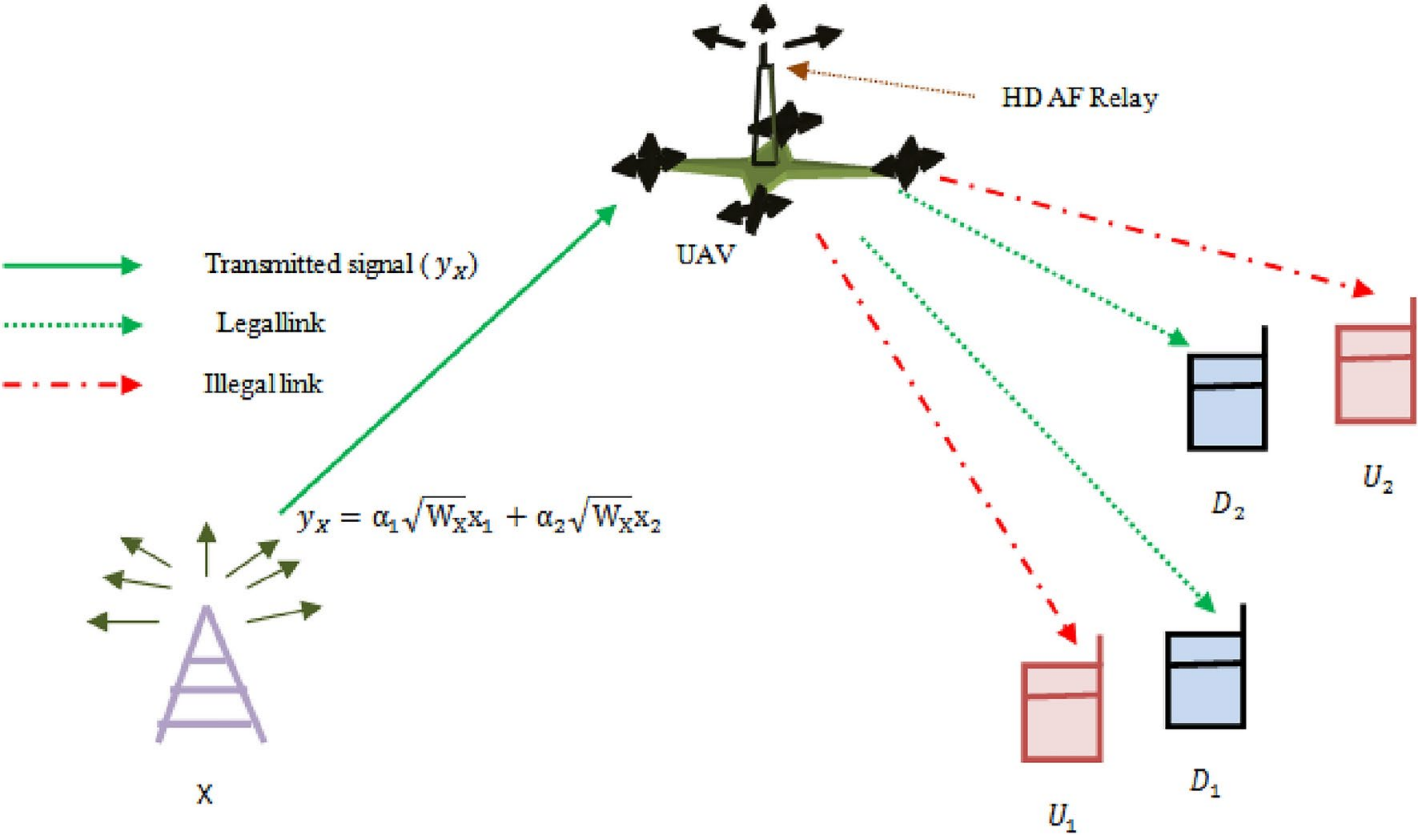
UAV assisted cooperative NOMA system with two untrusted destinations.

We consider a UAV-assisted cooperative Non-Orthogonal Multiple Access (NOMA) system consisting of a base station as the source (*X*), a half-duplex amplify-and-forward (HD-AF) relay (*R*) operating in half-duplex mode, two destinations ($D_1$, far destination, and $D_2$, near destination), and two untrusted destinations ($U_1$ and $U_2$). Figure 1 shows the UAV assisted cooperative NOMA system with two untrusted destinations.

The links between *X* to *R*, *R* to $D_1$, and *R* to $D_2$ are considered as legal links, while the links from the relay to $U_1$ and $U_2$ are considered as illegal links. All channels are assumed to suffer from independent Rayleigh fading, with the following channel gains and corresponding Rayleigh parameters: $h_X^R, \; h_R^{D_1}, \; h_R^{D_2}, \; h_R^{U_1}, \; \text {and} \; h_R^{U_2}$ with channel parameters $\lambda _X^R, \; \lambda _{R}^{D_1}, \; \lambda _{R}^{D_2}, \; \lambda _{R}^{U_1}, \; \text {and} \; \lambda _{R}^{U_2},$ respectively^60^.

Specifically, in the first time slot (FTSL), the source transmits a superposition of two different message signals, $x_{1}$ and $x_{2}$, to the relay R as follows^35^:

1 $$\begin{aligned} y_X = \alpha _{1} x_{1} \sqrt{W_{X}} + \alpha _{2} x_{2} \sqrt{W_{X}} \end{aligned}$$

where $x_i$ denotes the *i*-th data symbol for the *i*-th destination with normalized power $E[|x_i|^2] = 1$, $W_{X}$ is the total power transmitted from X, and $\alpha _i$ is the power allocation coefficient for the $i^{th}$ destination.

To fulfill the quality-of-service requirement of the first destination, we consider $\alpha _{1}$ to be greater than $\alpha _{2}$, and they satisfy the following expression: $\alpha _{1}^2 + \alpha _{2}^2 = 1.$ Then, the received signal $y_X^R$ at R in the FTSL can be expressed as:

2 $$\begin{aligned} y_X^R = h_X^R \left( \alpha _{1} x_{1} \sqrt{W_{X}} + \alpha _{2} x_{2} \sqrt{W_{X}} \right) + n_X^R, \end{aligned}$$

where $n_X^R$ represents the additive white Gaussian noise (AWGN) with zero mean and variance $N_D$.

In the second time slot (STSL), the AF relay amplifies the signal and forwards it to the destinations. The received signal at the *i*-th trusted destination can be expressed as:

3 $$\begin{aligned} y_{D_i}^R = \beta y_X^R h_R^{D_i} + n_{D_i}^R \end{aligned}$$

where $h_R^{D_i}$ is the channel gain between R and the *i*-th destination, $n_{D_i}^R$ denotes the additive Gaussian noise at the *i*-th destination with zero mean and variance $N_D$, and $\beta$ represents the amplification factor, which can be defined as:

4 $$\begin{aligned} \beta = \sqrt{\frac{W_{r}}{W_{X} |h_X^R|^2 + N_D}} \end{aligned}$$

where, $W_{r}$ is the relay transmitted power. Furthermore, after considering $W_{r}= W_{X}$, Eq. (4) can be simplified to:

5 $$\begin{aligned} \beta = \sqrt{\frac{1}{|h_X^R|^2 + \frac{1}{\rho _1}}} \end{aligned}$$

where, $\rho _1 = \frac{W_{X}}{N_D}$ is the average signal-to-noise ratio (SNR) of the legal link. Similarly, the signal received by the *i*-th untrusted destination can be expressed as:

6 $$\begin{aligned} y_{U_i}^R = \beta y_X^R h_{R}^{U_i} + n_{U_i}^R \end{aligned}$$

where $h_{R}^{U_i}$ is the channel gain between R and the *i*-th untrusted destination, and $n_{U_i}^R$ denotes the additive Gaussian noise with zero mean and variance $N_{U}$.

Substituting $y_X^R$ from Eq. (2) into Eq. (3), the received signal at the *i*-th trusted destination $y_{D_i}^R$ can be modified as:

7 $$\begin{aligned} y_{D_i}^R = \beta \Bigg [ h_X^R \left( \alpha _{1} x_{1} \sqrt{W_{X}} + \alpha _{2} x_{2} \sqrt{W_{X}} \right) + n_X^R \Bigg ] h_R^{D_i} + n_{D_i}^R \end{aligned}$$

The received signals at the trusted and untrusted destinations, $y_{D_1}^R$, $y_{D_2}^R$, $y_{U_1}^R$, and $y_{U_2}^R$, can be defined as:

8 $$\begin{aligned} y_{D_1}^R= & \beta h_X^R \alpha _{1} x_{1} \sqrt{W_{X}} h_R^{D_1} + \beta h_X^R \alpha _{2} x_{2} \sqrt{W_{X}} h_R^{D_1} + \beta n_X^R h_R^{D_1} + n_{D_1}^R \end{aligned}$$

9 $$\begin{aligned} y_{D_2}^R= & \beta h_X^R \alpha _{1} x_{1} \sqrt{W_{X}} h_R^{D_2} + \beta h_X^R \alpha _{2} x_{2} \sqrt{W_{X}} h_R^{D_2} + \beta n_X^R h_R^{D_2} + n_{D_2}^R \end{aligned}$$

10 $$\begin{aligned} y_{U_1}^R= & \left( \beta h_X^R \alpha _{1} x_{1} \sqrt{W_{X}} h_R^{U_1} + \beta h_X^R \alpha _{2} x_{2} \sqrt{W_{X}} h_R^{U_1} \right) + \beta n_X^R h_R^{U_1} + n_{U_1}^R \end{aligned}$$

11 $$\begin{aligned} y_{U_2}^R= & \left( \beta h_X^R \alpha _{1} x_{1} \sqrt{W_{X}} h_R^{U_2} + \beta h_X^R \alpha _{2} x_{2} \sqrt{W_{X}} h_R^{U_2} \right) + \beta n_X^R h_R^{U_2} + n_{U_2}^R \end{aligned}$$

The Signal-to-Interference-plus-Noise Ratio (SINR) at the far destination $D_1$, $\gamma _{D_1}$, can be defined using Eq. (8) as:

12 $$\begin{aligned} \gamma _{D_1} = \frac{\beta ^2 |h_X^R|^2 \alpha _{1}^2 W_{X} |h_R^{D_1}|^2}{\beta ^2 |h_X^R|^2 \alpha _{2}^2 W_{X} |h_R^{D_1}|^2 + \beta ^2 |n_X^R|^2 |h_R^{D_1}|^2 + |n_{D_1}^R|^2} \end{aligned}$$

After substituting $\beta$ from Eq. (5) into Eq. (12) and considering $\rho _1 = \frac{W_{X}}{N_D}$, Eq. (12) can be modified as:

The Signal-to-Interference-plus-Noise Ratio (SINR) at the far destination $D_1$ can be defined as:

13 $$\begin{aligned} \gamma _{D_1} = \frac{|h_X^R|^2 \alpha _{1}^2 |h_R^{D_1}|^2}{|h_X^R|^2 \alpha _{2}^2 |h_R^{D_1}|^2 + \frac{|h_R^{D_1}|^2}{\rho _1} + \frac{|h_X^R|^2}{\rho _1} + \frac{1}{\rho _1^2}} \end{aligned}$$

For the near destination $D_2$, which first decodes the message of $D_1$ and then performs perfect successive interference cancellation (SIC), the SNR can be defined as:

14 $$\begin{aligned} \gamma _{D_2} = \frac{|h_X^R|^2 \alpha _{2}^2 |h_R^{D_2}|^2}{\frac{|h_R^{D_2}|^2}{\rho _1} + \frac{|h_X^R|^2}{\rho _1} + \frac{1}{\rho _1^2}} \end{aligned}$$

For the eavesdropper at $U_1$, the SINR can be defined as:

15 $$\begin{aligned} \gamma _{U_1} = \frac{|h_X^R|^2 \alpha _{1}^2 |h_R^{U_1}|^2}{\frac{|h_R^{U_1}|^2}{\rho _1} + \frac{|h_X^R|^2}{\rho _2} + \frac{1}{\rho _1 \rho _2}} \end{aligned}$$

where $\rho _2 = \frac{W_{X}}{N_{U}}$ denotes the average SNR of the illegal link.

Similarly, the SNR at $U_2$ can be defined as:

16 $$\begin{aligned} \gamma _{U_2} = \frac{|h_X^R|^2 \alpha _{2}^2 |h_R^{U_2}|^2}{\frac{|h_R^{U_2}|^2}{\rho _1} + \frac{|h_X^R|^2}{\rho _2} + \frac{1}{\rho _1 \rho _2}} \end{aligned}$$

Considering the case of high SINR, the previous equations can be simplified as:

17 $$\begin{aligned} \gamma _{D_1}= & \frac{\alpha _{1}^2}{\alpha _{2}^2} \end{aligned}$$

18 $$\begin{aligned} \gamma _{D_2}= & \frac{\rho _1^2 |h_X^R|^2 \alpha _{2}^2 |h_R^{D_2}|^2}{\rho _1 |h_R^{D_2}|^2 + \rho _1 |h_X^R|^2} \end{aligned}$$

19 $$\begin{aligned} \gamma _{U_1}= & \frac{\rho _1 \rho _2 |h_X^R|^2 \alpha _{1}^2 |h_R^{U_1}|^2}{\rho _2 |h_R^{U_1}|^2 + \rho _1 |h_X^R|^2} \end{aligned}$$

20 $$\begin{aligned} \gamma _{U_2}= & \frac{\rho _1 \rho _2 |h_X^R|^2 \alpha _{2}^2 |h_R^{U_2}|^2}{\rho _2 |h_R^{U_2}|^2 + \rho _1 |h_X^R|^2} \end{aligned}$$

The average rate from the relay to trusted destinations $C_{R}^{D_i}$ and untrusted destinations $C_{R}^{U_i}$ can be expressed as:

21 $$\begin{aligned} C_{R}^{D_i}= & \frac{1}{2} \log _2(1 + \gamma _{D_i}) \end{aligned}$$

22 $$\begin{aligned} C_{R}^{U_i}= & \frac{1}{2} \log _2(1 + \gamma _{U_i}) \end{aligned}$$

In the proposed system model, $U_1$ decodes the message of $D_1$ and $U_2$ decodes the message of $D_2$. Thus, the secrecy rates for $D_1$ and $D_2$ can be defined as:

23 $$\begin{aligned} C_{D_1}^{S}= & |C_{R}^{D_1} - C_{R}^{U_1}|^+ \end{aligned}$$

24 $$\begin{aligned} C_{D_2}^{S}= & |C_{R}^{D_2} - C_{R}^{U_2}|^+ \end{aligned}$$

## Secrecy performance analysis

In this section, we first derive the analytical expressions for the SOP and SPSC for the proposed cooperative UAV-assisted cooperative NOMA system with two untrusted destinations over Rayleigh fading.

### Secrecy outage probability

The Secrecy Outage Probability (SOP) is defined as the probability that the instantaneous Signal-to-Noise Ratio (SNR) is lower than a specified threshold^61–63^. In the proposed system model, two message signals are sent from the source (*X*) to the far destination $(D_1)$ and the near destination $(D_2)$ respectively, through a relay. An outage occurs either when $\gamma _{D_1}$ or $\gamma _{D_2}$ starts to fall below their respective target rates ($R_1$ or $R_2$). The SOP can be represented using this definition as:

25 $$\begin{aligned} \text {SOP}_{AF} = P\left[ C_{D_1}^S< R_1, C_{D_2}^S < R_2 \right] \end{aligned}$$

where $P[\cdot ]$ measures the probability. Equation (25) can be modified using Eqs. (23) and (24) as:

26 $$\begin{aligned} \text {SOP}_{AF} = P\left[ C_{R}^{D_1} - C_{R}^{U_1}< R_1, C_{R}^{D_2} - C_{R}^{U_2} < R_2 \right] \end{aligned}$$

Equation (26) can be further modified using Eqs. (21) and (22) as:

27 $$\begin{aligned} \text {SOP}_{AF} = P \Bigg [ \Big (\frac{1}{2} \log _2(1+\gamma _{D_1}) - \frac{1}{2} \log _2(1+\gamma _{U_1})< R_1\Big ), \Big (\frac{1}{2} \log _2(1+\gamma _{D_2}) - \frac{1}{2} \log _2(1+\gamma _{U_2}) < R_2 \Big )\Bigg ] \end{aligned}$$

We can express the SOP as:

28 $$\begin{aligned} \text {SOP}_{AF} = 1 - P\left[ \frac{(1+\gamma _{D_1})}{(1+\gamma _{U_1})}> C_{th}^{'}, \frac{(1+\gamma _{D_2})}{(1+\gamma _{U_2})} > C_{th}^{''} \right] \end{aligned}$$

where $C_{th}^{'} = 2^{2R_1}$ and $C_{th}^{''} = 2^{2R_2}$. Eq. (28) can be modified using Eqs. (17) and (19) as^64^:

29 $$\begin{aligned} \text {SOP}_{AF} = 1 - P\Bigg [ \frac{\rho _2 |h_R^{U_1}|^2 + \rho _1 |h_X^R|^2 + \rho _1 \rho _2 |h_X^R|^2 \alpha _{1}^2 |h_R^{U_1}|^2}{\rho _2 |h_R^{U_1}|^2 + \rho _1 |h_X^R|^2} < \frac{1}{\alpha _{2}^2 C_{th}^{'}}, \gamma _{D_2} > C_{th}^{''} + C_{th}^{''} \gamma _{U_2}-{1} \Bigg ] \end{aligned}$$

Equation (29) can be modified using Eqs. (18) and (20) as:

30 $$\begin{aligned} \text {SOP}_{AF} = 1 - P\Bigg [ \frac{\rho _1 \rho _2 |h_X^R|^2 |h_R^{U_1}|^2}{\rho _2 |h_R^{U_1}|^2 + \rho _1 |h_X^R|^2} < L_1, \frac{\rho _1^2 |h_X^R|^2 \alpha _{2}^2 |h_R^{D_2}|^2}{\rho _1 |h_R^{D_2}|^2 + \rho _1 |h_X^R|^2} > C_{th}^{''} + C_{th}^{''} \frac{\rho _1 \rho _2 |h_X^R|^2 \alpha _{2}^2 |h_R^{U_2}|^2}{\rho _2 |h_R^{U_2}|^2 + \rho _1 |h_X^R|^2} - 1 \Bigg ] \end{aligned}$$

where $L_1 = \frac{1 - \alpha _{2}^2 C_{th}^{'}}{\alpha _{1}^2 \alpha _{2}^2 C_{th}^{'}}$. Here $L_1$ should be greater than zero; otherwise, the SOP will be one.

Now, after applying the broad equality^65^, i.e., $\frac{ab}{a+b} \le \min \{a,b\}$ in Eq. (30), we can modify Eq. (30) as:

31 $$\begin{aligned} \text {SOP}_{AF} \le 1 - P\Big (\min \Big \{\rho _1 |h_{X}^{R}|^2, \rho _2 |h_{R}^{U_1}|^2 \Big \} < L_1, \min \Big \{|h_{X,R}|^2, |h_R^{D_2}|^2\Big \} > \Big \{C_{\text {th}}'' |h_X^R|^2, A' |h_R^{U_2}|^2\Big \} + B'\Big ) \end{aligned}$$

where $A^{'} = \frac{\rho _2}{\rho _1} C_{th}^{''}$ and $B^{'} = \frac{C_{th}^{''}-1}{\rho _1 \alpha _{2}^2}$. As $P(l_1, l_2) = P(l_1) - P\left( l_1, \overline{l_2}\right)$^65^, now considering:

$$\begin{aligned} l_1&= \min \left\{ |h_X^R|^2, |h_R^{D_2}|^2\right\} > \min \left\{ C_{th}^{''} |h_X^R|^2, A^{'} |h_R^{U_2}|^2\right\} + B^{'} \\ l_2&= \min \left\{ \rho _1 |h_X^R|^2, \rho _2 |h_R^{U_1}|^2\right\} < L_1 \end{aligned}$$

in Eq. (31), it can be modified as:

32 $$\begin{aligned} \text {SOP}_{AF}= & 1 - \Bigg [ P\Big (\min \Big \{|h_X^R|^2, |h_R^{D_2}|^2\Big \}> \min \Big \{C_{th}^{''} |h_X^R|^2, A^{'} |h_R^{U_2}|^2\Big \} + B^{'}\Big ) \nonumber \\ & - P\Big (\min \Big \{|h_X^R|^2, |h_R^{D_2}|^2\Big \}> \min \Big \{C_{th}^{''} |h_X^R|^2, A^{'} |h_R^{U_2}|^2\Big \} + B^{'}, \min \Big \{\rho _1 |h_X^R|^2, \rho _2 |h_R^{U_1}|^2\Big \} > L_1 \Big ) \Bigg ] \end{aligned}$$

Let $x^{'} = |h_R^{U_2}|^2$ and $|h_X^R|^2$ be smaller than $C_{th}^{''} |h_X^R|^2 + B^{'}$. So, Eq. (32) can be modified as:

The SOP for the proposed system can be calculated from the above expression as follows:

33 $$\begin{aligned} \text {SOP}_{AF} = 1 - \left[ P' - P''\right] \end{aligned}$$

where, $P'=P\Big ( |h_X^R|^2> A'x' + B', |h_{R}^{D_2}|^2 > A'x' + B' \Big )$ and $P''=P\Big \{|h_X^R|^2> (A'x' + B'), |h_{R}^{D_2}|^2> (A'x' + B') \Big \} P\Big (\rho _1 |h_X^R|^2, \rho _2 |h_{R}^{D_1}|^2 > L_1\Big )$. Now considering $P'$:

34 $$\begin{aligned} P' = P\left( |h_X^R|^2> A'x' + B', |h_{R}^{D_2}|^2 > A'x' + B' \right) \end{aligned}$$

As $$P\left( |h_X^R|^2 > A'x' + B' \right) = 1 - \int _0^{A'x' + B'} \lambda _X^R e^{-\lambda _X^R y} \, dy = e^{-\lambda _X^R(A'x' + B')}$$ and $$P\left( |h_{R}^{D_2}|^2 > A'x' + B' \right) = e^{-\lambda _{R}^{D_2}(A'x' + B')}.$$

So Eq. (34) can be modified as:

35 $$\begin{aligned} P'= & \int _0^\infty e^{-\lambda _X^R(A'x' + B')} e^{-\lambda _{R}^{D_2}(A'x' + B')} f_{|h_{R}^{U_2}|^2}(x') \, dx' \end{aligned}$$

36 $$\begin{aligned} P'= & \int _0^\infty e^{-\lambda _X^R(A'x' + B')} e^{-\lambda _{R}^{D_2}(A'x' + B')} \lambda _{R}^{U_2} e^{-\lambda _{R}^{U_2} x'} \, dx' \end{aligned}$$

37 $$\begin{aligned} P'= & \frac{\lambda _{R}^{U_2} e^{-(\lambda _X^R + \lambda _{R}^{D_2})B'}}{\lambda _X^R A' + \lambda _{R}^{D_2} A' + \lambda _{R}^{U_2}} \end{aligned}$$

Now considering $$P''$$:

38 $$\begin{aligned} P'' = P\left( |h_X^R|^2> A'x' + B', |h_{R}^{D_2}|^2> A'x' + B' \right) \times P\left( |h_X^R|^2> \frac{L_1}{\rho _1} \right) \times P\left( |h_{R}^{U_1}|^2 > \frac{L_1}{\rho _2} \right) \end{aligned}$$

Similar to Eqs. (34), (38)) can be modified as:

39 $$\begin{aligned} P''= & \left( \int _0^\infty e^{-\lambda _X^R(A'x' + B')} e^{-\lambda _{R}^{D_2}(A'x' + B')} f_{|h_{R}^{U_2}|^2}(x') \, dx' \right) \times \left[ 1 - P\left( |h_X^R|^2< \frac{L_1}{\rho _1} \right) \times \left( 1 - P\left( |h_{R}^{U_1}|^2 < \frac{L_1}{\rho _2} \right) \right) \right] \end{aligned}$$

40 $$\begin{aligned} P''= & \left( \int _0^\infty e^{-\lambda _X^R(A'x' + B')} e^{-\lambda _{R}^{D_2}(A'x' + B')} \lambda _{R}^{U_2} e^{-\lambda _{R}^{U_2} x'} \, dx' \right) \times \left( 1 - \int _0^{\frac{L_1}{\rho _1}} e^{-\lambda _X^R x'} \, dx' \right) \times \left( 1 - \int _0^{\frac{L_1}{\rho _2}} e^{-\lambda _{R}^{U_1} x'} \, dx' \right) \end{aligned}$$

41 $$\begin{aligned} P''= & \frac{\lambda _{R}^{U_2} e^{-(\lambda _X^R + \lambda _{R}^{D_2})B'}}{\lambda _X^R A' + \lambda _{R}^{D_2} A' + \lambda _{R}^{U_2}} \times e^{-\lambda _X^R \frac{L_1}{\rho _1}} \times e^{-\lambda _{R}^{U_1} \frac{L_1}{\rho _2}} \end{aligned}$$

Now, substituting the values of $P'$ and $P''$ into Eq. (33):

42 $$\begin{aligned} \text {SOP}^{AF} = 1 - \frac{\lambda _{R}^{U_2} e^{-(\lambda _X^R + \lambda _{R}^{D_2})B'}}{\lambda _X^R A' + \lambda _{R}^{D_2} A' + \lambda _{R}^{U_2}} + \frac{\lambda _{R}^{U_2} e^{-(\lambda _X^R + \lambda _{R}^{D_2})B'}}{\lambda _X^R A' + \lambda _{R}^{D_2} A' + \lambda _{R}^{U_2}} \times e^{-\lambda _X^R \frac{L_1}{\rho _1}} \times e^{-\lambda _{R}^{U_1} \frac{L_1}{\rho _2}} \end{aligned}$$

The final expression of SOP contains exponential terms, which indicates that it depends upon channel coefficient gains, power allocation coefficient, and target rates. These parameters need to be optimized for optimizing wireless systems under security constraints.

### Strictly positive secrecy capacity

The Secrecy Positive Secrecy Capacity (SPSC) is a fundamental benchmark in secure communications. SPSC measures the maximum achievable secrecy rate for reliable communication and ensures that eavesdroppers cannot decode the messages. With this description, the SPSC may be defined as^65^:

43 $$\begin{aligned} \text {SPSC}^{AF} = P(C_{D_1}^{AF}> 0, C_{D_2}^{AF} > 0) \end{aligned}$$

Using Eqs. (21) to (24), Eq. (43) can be modified as:

44 $$\begin{aligned} \text {SPSC}^{AF} = P(\gamma _{D_1}> \gamma _{U_1}, \gamma _{D_2} > \gamma _{U_2}) \end{aligned}$$

Using Eqs. (17) to (20), Eq. (44) can be further modified as:

45 $$\begin{aligned} \text {SPSC}^{AF} = P\left( \frac{\alpha _{1}^2}{\alpha _{2}^2}> \frac{\rho _1 \rho _2 |h_X^R|^2 \alpha _{1}^2 |h_R^{U_1}|^2}{\rho _2 |h_R^{U_1}|^2 + \rho _1 |h_X^R|^2}, \right. \left. \frac{\rho _1^2 |h_X^R|^2 \alpha _{2}^2 |h_R^{D_2}|^2}{\rho _1 |h_R^{D_2}|^2 + \rho _1 |h_X^R|^2} > \frac{\rho _1 \rho _2 |h_X^R|^2 \alpha _{2}^2 |h_R^{U_2}|^2}{\rho _2 |h_R^{U_2}|^2 + \rho _1 |h_X^R|^2} \right) \end{aligned}$$

Now, after applying the broad equality^65^ in Eq. (45), it can be modified as:

46 $$\begin{aligned} \text {SPSC}^{AF} = P\left( \frac{\alpha _{1}^2}{\alpha _{2}^2}> \min \left\{ \rho _1 |h_X^R|^2, \rho _2 |h_R^{U_1}|^2\right\} \alpha _{1}^2, \right. \left. \min \left\{ \rho _1 |h_X^R|^2, \rho _1 |h_R^{D_2}|^2\right\} > \min \left\{ \rho _1 |h_X^R|^2, \rho _2 |h_R^{U_2}|^2\right\} \right) \end{aligned}$$

Equation (46) can then be expressed as:

47 $$\begin{aligned} \text {SPSC}^{AF}= & P\left( \min \left\{ \rho _1 |h_X^R|^2, \rho _1 |h_R^{D_2}|^2\right\}> \min \left\{ \rho _1 |h_X^R|^2, \rho _2 |h_R^{U_2}|^2\right\} \right) \nonumber \\ & - P\left( \min \left\{ \rho _1 |h_X^R|^2, \rho _1 |h_R^{D_2}|^2\right\} > \min \left\{ \rho _1 |h_X^R|^2, \rho _2 |h_R^{U_2}|^2\right\} , \right. \left. \frac{\alpha _{1}^2}{\alpha _{2}^2} < \min \left\{ \rho _1 |h_X^R|^2, \rho _2 |h_R^{U_1}|^2\right\} \alpha _{1}^2\right) \end{aligned}$$

Further simplification gives:

48 $$\begin{aligned} \text {SPSC}^{AF}= & P\left( \min \left\{ \rho _1 |h_X^R|^2, \rho _1 |h_{R}^ {D_2}|^2\right\}> \rho _2 |h_{R}^ {U_2}|^2\right) - P\Big (\min \left\{ \rho _1 |h_X^R|^2, \rho _1 |h_{R}^ {D_2}|^2\right\} > \rho _2 |h_{R}^ {U_2}|^2, \frac{1}{\alpha _{2}^2} < \rho _2 |h_{R}^ {U_1}|^2\Big ) \end{aligned}$$

49 $$\begin{aligned} \text {SPSC}^{AF}= & P\left( |h_X^R|^2> \frac{\rho _2}{\rho _1} |h_R^{U_2}|^2, |h_R^{D_2}|^2> \frac{\rho _2}{\rho _1} |h_R^{U_2}|^2 \right) - P\Big ( |h_X^R|^2> \frac{\rho _2}{\rho _1} |h_R^{U_2}|^2, |h_R^{D_2}|^2 > \frac{\rho _2}{\rho _1} |h_R^{U_2}|^2, \frac{1}{\rho _2 \alpha _{2}^2} < |h_R^{U_1}|^2 \Big ) \end{aligned}$$

Letting $x' = |h_R^{U_2}|^2$, Eq. (49) becomes:

50 $$\begin{aligned} \text {SPSC}^{AF} = \int _0^{\infty } e^{-\lambda _X^R \frac{\rho _2}{\rho _1} x'} e^{-\lambda _{R}^{D_2} \frac{\rho _2}{\rho _1} x'} \lambda _{R}^{U_2} e^{-\lambda _{R}^{U_2} x'} \, dx' - \int _0^{\infty } e^{-\lambda _X^R \frac{\rho _2}{\rho _1} x'} e^{-\lambda _{R}^{D_2} \frac{\rho _2}{\rho _1} x'} \lambda _{R}^{U_2} e^{-\lambda _{R}^{U_2} x'} \, dx' \cdot e^{-\frac{\lambda _{R}^{U_1}}{\rho _2 \alpha _{2}^2}} \end{aligned}$$

After further simplification, we obtain:

51 $$\begin{aligned} \text {SPSC}^{AF} = \Bigg [\frac{\lambda _{R}^{U_2}}{\lambda _X^R \frac{\rho _2}{\rho _1} + \lambda _{R}^{D_2} \frac{\rho _2}{\rho _1} + \lambda _{R}^{U_2}} - \frac{\lambda _{R}^{U_2} \cdot e^{-\frac{\lambda _{R}^{U_1}}{\rho _2 \alpha _{2}^2}}}{\lambda _X^R \frac{\rho _2}{\rho _1} + \lambda _{R}^{D_2} \frac{\rho _2}{\rho _1} + \lambda _{R}^{U_2}}\Bigg ] \end{aligned}$$

which can be written as:

52 $$\begin{aligned} \text {SPSC}^{AF} = \frac{\lambda _{R}^{U_2}}{\lambda _X^R \frac{\rho _2}{\rho _1} + \lambda _{R}^{D_2} \frac{\rho _2}{\rho _1} + \lambda _{R}^{U_2}} \left[ 1 - e^{-\frac{\lambda _{R}^{U_1}}{\rho _2 \alpha _{2}^2}} \right] \end{aligned}$$

The final expression of SPSC depends upon channel coefficient gains and ratio of SNRs of illegal and legal links.

### Asymptotic SOP analysis

From Eq. (28), we have:

$$\begin{aligned} & \gamma _{U_1} \approx \rho _2 \alpha _{1}^2 |h_{R} ^{U_1}|^2, \quad \gamma _{UD_2} \approx \rho _2 \alpha _{2}^2 |h_{R}^ {U_2}|^2, \quad \gamma _{D_1} \approx \frac{\alpha _{1}^2}{\alpha _{2}^2}, \\ & \quad \text {and} \quad \gamma _{D_2} \approx \infty \quad \text {when } \rho _1 \rightarrow \infty . \end{aligned}$$

Therefore, the SOP performance of the proposed AF-based NOMA systems can be asymptotically expressed as:

53 $$\begin{aligned} & \text {SOP}_{AF} = 1 - P\left( \frac{1 + \gamma _{D_1}}{1 + \gamma _{U_1}} > C_{th}' \right) \end{aligned}$$

54 $$\begin{aligned} & \text {SOP}_{AF} = P\left( \frac{1 + \frac{\alpha _{1}^2}{\alpha _{2}^2}}{1 + \rho _2 \alpha _{1}^2 |h_{R} ^{U_1}|^2} < C_{th}' \right) \end{aligned}$$

Since $\alpha _{1}^2 + \alpha _{2}^2 = 1$, Eq. (54) can be modified as:

55 $$\begin{aligned} \text {SOP}_{AF}= & P\left( \frac{1}{\alpha _{2}^2 C_{th}'} < 1 + \rho _2 \alpha _{1}^2 |h_{R} ^{U_1}|^2 \right) \end{aligned}$$

56 $$\begin{aligned} \text {SOP}_{AF}= & P\left( \frac{1 - \alpha _{2}^2 C_{th}'}{\rho _2 \alpha _{1}^2 \alpha _{2}^2 C_{th}'} < |h_{R} ^{U_1}|^2 \right) \end{aligned}$$

Letting $L_1 = \frac{1 - \alpha _{2}^2 C_{th}'}{\alpha _{1}^2 \alpha _{2}^2 C_{th}'}$, Eq. (56) can be modified as:

57 $$\begin{aligned} \text {SOP}_{AF} = P\left( \frac{L_1}{\rho _2} < |h_{R} ^{U_1}|^2 \right) \end{aligned}$$

Thus, the final expression for SOP is:

58 $$\begin{aligned} \text {SOP}_{AF} = e^{-\frac{\lambda _{R} ^{U_1} \times L_1}{\rho _2}} \end{aligned}$$

Equation (58) represents the asymptotic expression of the SOP for the proposed UAV-assisted cooperative NOMA system in presence of eavesdroppers. This equation shows that SOP depends on the Rayleigh fading channel parameter of the illegal link between *R* to $U_1$, ratio of the legal to illegal SNR $(\rho _2)$ and $L_1$ which further depends upon power allocation coefficients $(\alpha _{1}, \alpha _{2})$ and target secrecy rate (*R*1).

Therefore, the final expressions of SOP and SPSC contain exponential terms and depend on channel coefficient gains, power allocation coefficients, and target rates.

## Numerical results

In this section, we provide the simulation results to show the secrecy performance of the proposed UAV-assisted NOMA-based cooperative system with two untrusted destinations. We use Monte Carlo simulations to validate the analytical results.

**Figure 2:**
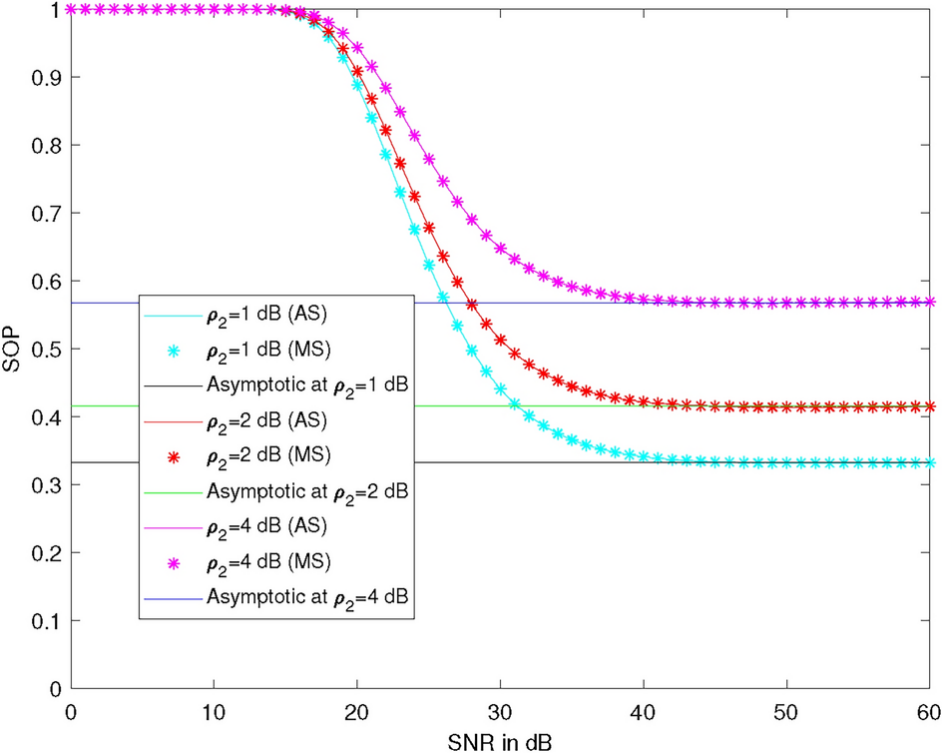
SOP vs SNR at different average illegal SNR.

Figure 2 illustrates the plots of SOP versus SNR at different values of average illegal SNR ($\rho _2$), specifically at 1 dB, 2 dB, and 4 dB. Here, we take $\lambda _{R}^{U_2} = 1$, $\lambda _{R}^{U_1} = 1$, $\lambda _X^R = 1$, $\lambda _R^{D_2} = 1$, the power of the first allocation factor = 0.87, and $R_1 = 0.51$ bits per channel use (BPCU), $R_2 = 2.1$ BPCU. At 30 dB SNR, the SOP values are 0.44, 0.51, and 0.65 at $\rho _2 = 1$ dB, 2 dB, and 4 dB, respectively. This indicates that SOP is 16% more at $\rho _2 = 2$ dB and 48% more at $\rho _2 = 4$ dB compared to SOP at $\rho _2 = 1$ dB. Therefore, it is observed that SOP increases as the value of $\rho _2$ increases and decreases as $\rho _1$ increases up to 45 dB, becoming constant at larger values of $\rho _1$ (asymptotic SOP). Thus, SOP depends only on $\rho _2$ at large values of $\rho _1$, and small values of $\rho _2$ are required for reliable communication.

**Figure 3:**
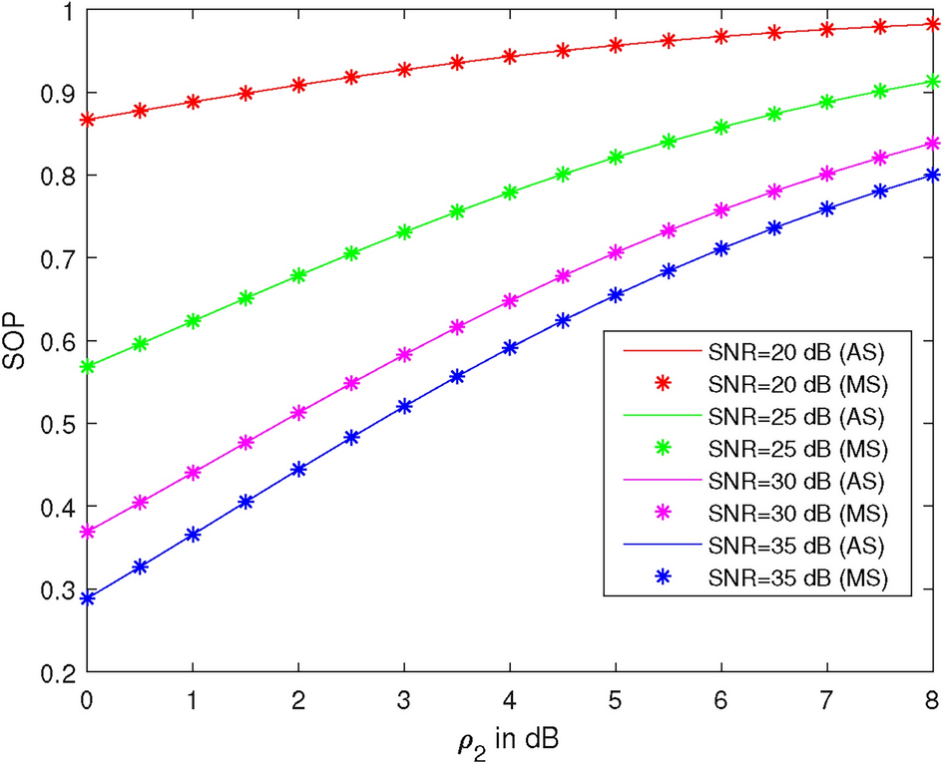
SOP vs average illegal SNR at different values of legal SNR.

Figure 3 shows the plots of SOP versus average illegal SNR ($\rho _2$) at different values of legal SNR ($\rho _1$), specifically at 20 dB, 25 dB, 30 dB, and 35 dB. The parameters used are $\lambda _{R}^{U_2} = 1$, $\lambda _{R}^{U_1} = 1$, $\lambda _X^R = 1$, $\lambda _R^{D_2} = 1$, power of the first allocation factor = 0.87, and $R_1 = 0.51$ BPCU, $R_2 = 2.1$ BPCU. At 4 dB average illegal SNR, the SOP values are 0.943, 0.779, 0.648, and 0.591 at legal SNRs of 20 dB, 25 dB, 30 dB, and 35 dB, respectively. Thus, SOP is 17% less at 25 dB, 31% less at 30 dB, and 37% less at 35 dB compared to SOP at 20 dB. It is observed that SOP increases as $\rho _2$ increases and decreases as $\rho _1$ increases.

**Figure 4:**
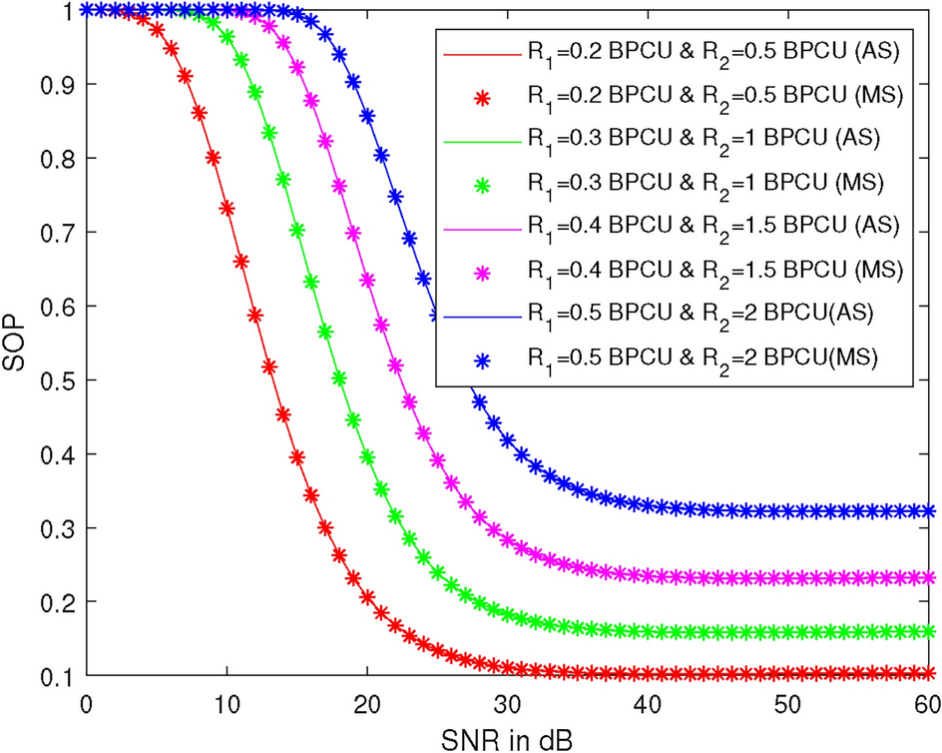
SOP vs SNR at different target rates.

Figure 4 represents the plots of SOP versus SNR at different target rates ($R_1$ and $R_2$), specifically $R_1 = 0.2$, 0.3, 0.4, and 0.5 BPCU, and $R_2 = 0.5$, 1, 1.5, and 2 BPCU. Here, we take $\lambda _{R}^{U_2} = 1$, $\lambda _{R}^{U_1} = 1$, $\lambda _X^R = 1$, $\lambda _R^{D_2} = 1$, power of the first allocation factor = 0.87, and $\rho _2 = 1$ dB. It is observed that SOP increases as the values of the target rate increase and decreases as $\rho _1$ increases.

**Figure 5:**
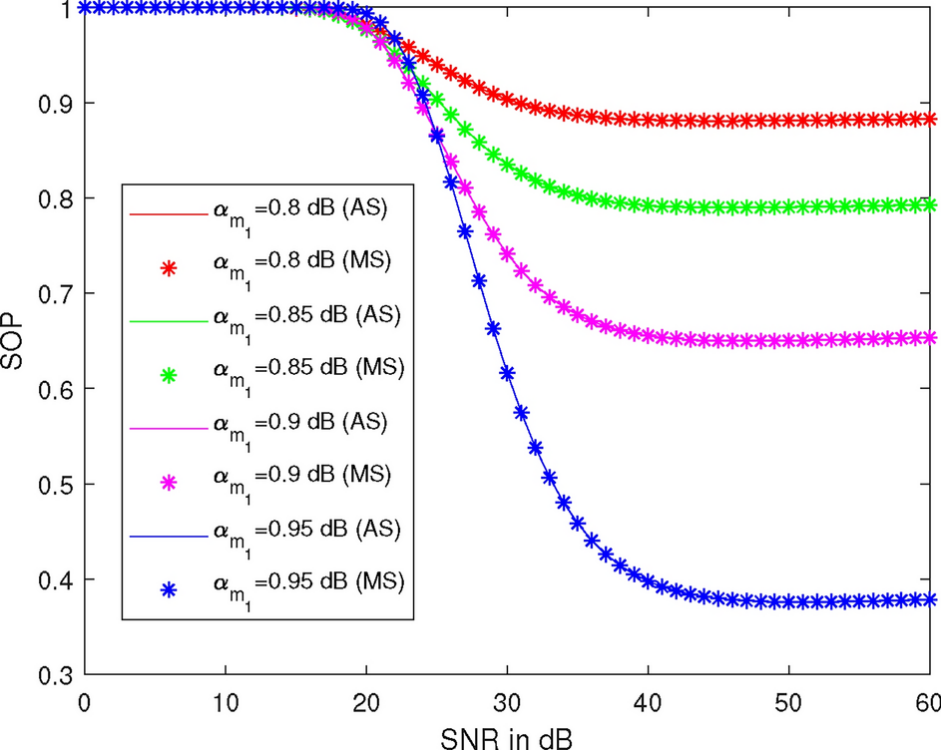
SOP vs SNR at different values of power allocation coefficient $\alpha _{1}$.

**Figure 6:**
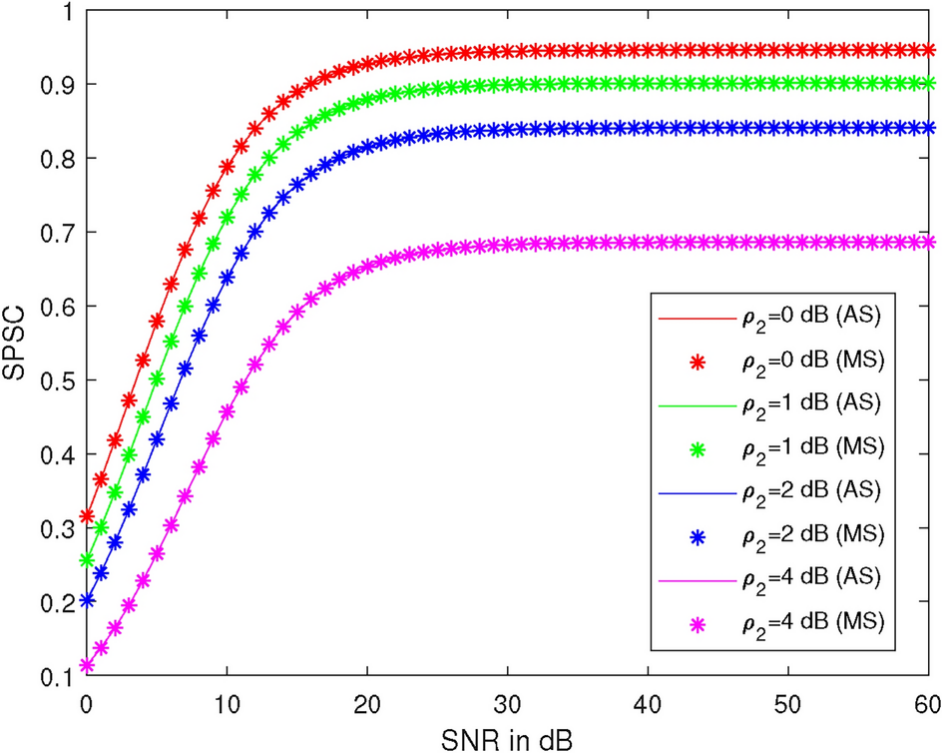
SPSC vs SNR at different values of average illegal SNRs.

Figure 5 illustrates the plots of SOP versus SNR at different values of the power allocation coefficient $\alpha _{1}$, specifically $\alpha _{1} = 0.8$, 0.85, 0.90, and 0.95. Here, we take $R_1 = 0.51$ BPCU, $R_2 = 2.1$ BPCU, $\lambda _{R}^{U_2} = 1$, $\lambda _{R}^{U_1} = 1$, $\lambda _X^R = 1$, $\lambda _R^{D_2} = 1$, power of the first allocation factor = 0.87, and $\rho _2 = 7$ dB. At 30 dB SNR, the SOP values are 0.90, 0.84, 0.74, and 0.62 at $\alpha _{1} = 0.8$, 0.85, 0.90, and 0.95, respectively. Thus, SOP is 6% less at $\alpha _{1} = 0.85$, 17% less at $\alpha _{1} = 0.90$, and 31% less at $\alpha _{1} = 0.95$ compared to SOP at $\alpha _{1} = 0.80$. Therefore, it is observed that SOP decreases as the values of $\alpha _{1}$ increase and decreases as $\rho _1$ increases.

Figure 6 shows the plots of SPSC versus SNR at different values of average illegal SNR ($\rho _2$), specifically at 0 dB, 1 dB, 2 dB, and 4 dB. Here, we take $\lambda _{R}^{U_2} = 1$, $\lambda _{R}^{U_1} = 1$, $\lambda _X^R = 1$, $\lambda _R^{D_2} = 1$, power of the first allocation factor = 0.81, $R_1 = 0.51$ BPCU, and $R_2 = 2.1$ BPCU. At 30 dB SNR, the SPSC values are 0.94, 0.90, 0.84, and 0.68 at $\rho _2 = 0$ dB, 1 dB, 2 dB, and 4 dB, respectively. Thus, SPSC is 4% less at $\rho _2 = 1$ dB, 16% less at $\rho _2 = 2$ dB, and 27% less at $\rho _2 = 4$ dB compared to SPSC at $\rho _2 = 0$ dB. Therefore, it is observed that SPSC decreases as $\rho _2$ increases and also increases with $\rho _1$ up to 20 dB SNR.

**Figure 7:**
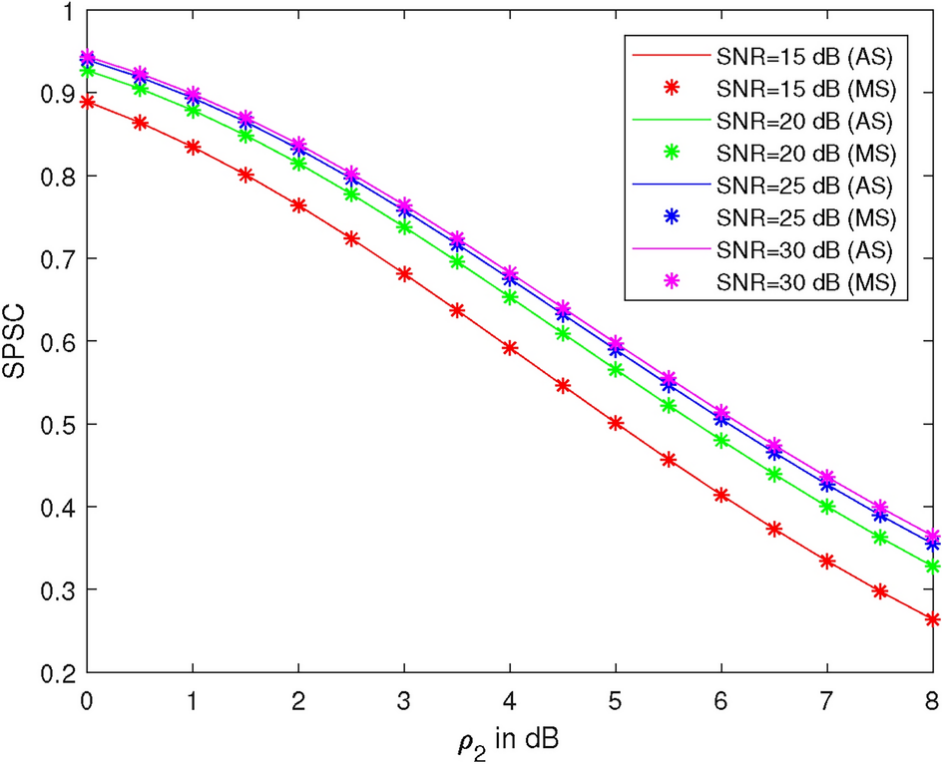
SPSC vs average illegal SNR at different values of legal SNR.

Figure 7 illustrates the plots of SPSC versus average illegal SNR ($\rho _2$) at different values of legal SNR ($\rho _1$), specifically at 15 dB, 20 dB, 25 dB, and 30 dB. Here, we take $\lambda _{R}^{U_2} = 1$, $\lambda _{R}^{U_1} = 1$, $\lambda _X^R = 1$, $\lambda _R^{D_2} = 1$, power of the first allocation factor = 0.81, $R_1 = 0.51$ BPCU, and $R_2 = 2.1$ BPCU. At 4 dB average illegal SNR, the SPSC values are 0.86, 0.79, 0.66, and 0.58 at legal SNRs of 15 dB, 20 dB, 25 dB, and 30 dB, respectively. Thus, SPSC is 7% less at 20 dB, 23% less at 25 dB, and 32% less at 30 dB compared to SPSC at 15 dB. It is observed that SPSC increases with $\rho _2$ and decreases as $\rho _1$ increases.

**Figure 8:**
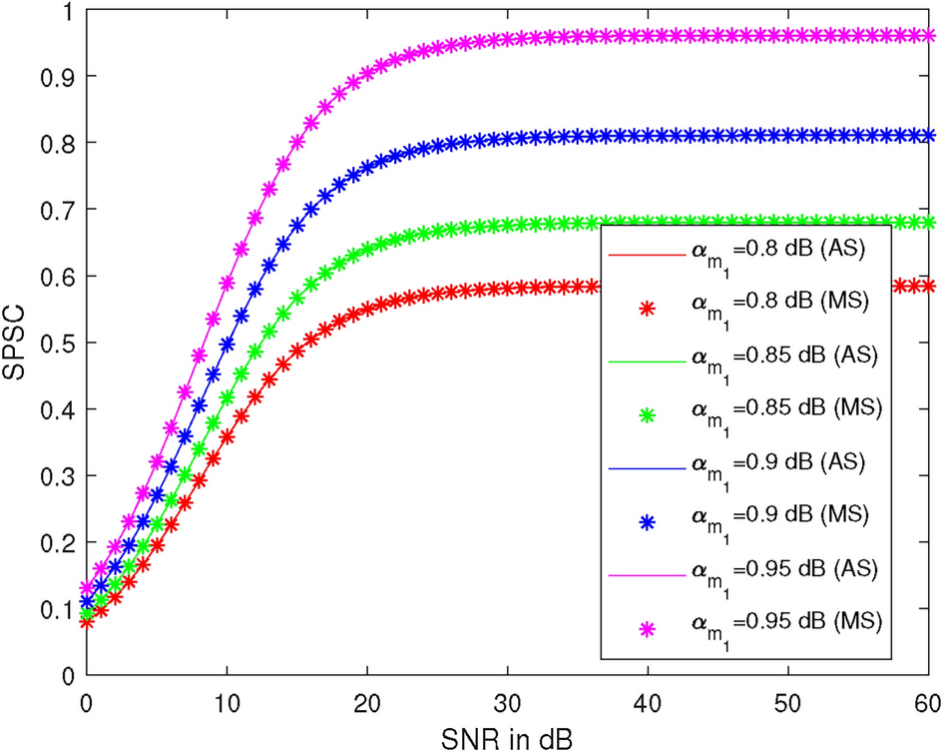
SPSC vs SNR at different values of power allocation coefficient $\alpha _{1}$.

Figure 8 represents the plots of SPSC versus SNR at different values of the power allocation coefficient $\alpha _{1}$, specifically $\alpha _{1} = 0.8, 0.85, 0.90, \text {and } 0.95$. Here, we take $R_1 = 0.51$ BPCU, $R_2 = 2.1$ BPCU, $\lambda _R^{U_2} = 1$, $\lambda _{R} ^{U_1} = 1$, $\lambda _X^R = 1$, $\lambda _R^{D_2} = 1$, and $\rho _2 = 7$ dB. At 30 dB SNR, the SPSC values are 0.58, 0.68, 0.81, and 0.96 at $\alpha _{1} = 0.8, 0.85, 0.90, \text {and } 0.95$, respectively. SPSC increases by 17% at $\alpha _{1} = 0.85$, 40% at $\alpha _{1} = 0.90$, and 65% at $\alpha _{1} = 0.95$ compared to $\alpha _{1} = 0.80$. It is observed that SPSC increases as the value of $\alpha _{1}$ increases and also with $\rho _1$ up to 20 dB.

**Figure 9:**
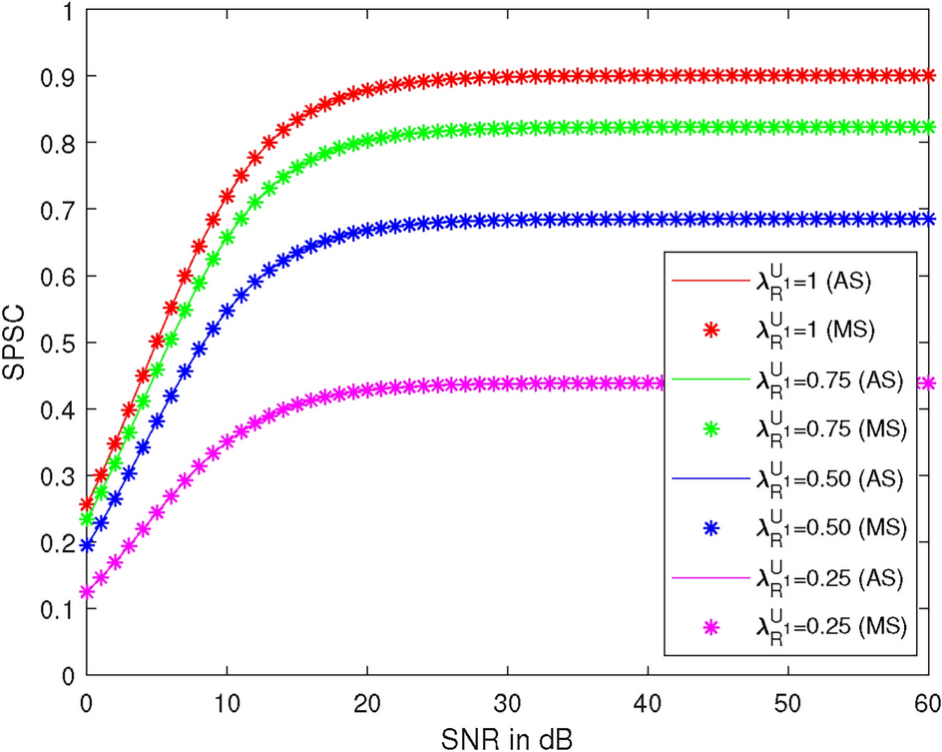
SPSC vs SNR at different values of $\lambda _{R} ^{U_1}$.

Figure 9 shows the plots of SPSC versus SNR at different values of $\lambda _{R} ^{U_1}$, specifically $1, 0.75, 0.50, \text {and } 0.25$. Here, we take $R_1 = 0.51$ BPCU, $R_2 = 2.1$ BPCU, $\lambda _R^{U_2} = 1$, $\lambda _X^R = 1$, $\lambda _R^{D_2} = 1$, the power of the first allocation factor = 0.87, and $\rho _2 = 1$ dB. At 30 dB SNR, the SPSC values are 0.90, 0.82, 0.68, and 0.44 at $\lambda _{R} ^{U_1} = 1, 0.75, 0.50, \text {and } 0.25$, respectively. SPSC decreases by 8% at $\lambda _{R} ^{U_1} = 0.75$, 24% at $\lambda _{R} ^{U_1} = 0.50$, and 51% at $\lambda _{R} ^{U_1} = 0.25$ compared to $\lambda _{R} ^{U_1} = 1$. It is observed that SPSC increases as $\lambda _{R} ^{U_1}$ increases and also with $\rho _1$ up to 20 dB.

**Figure 10:**
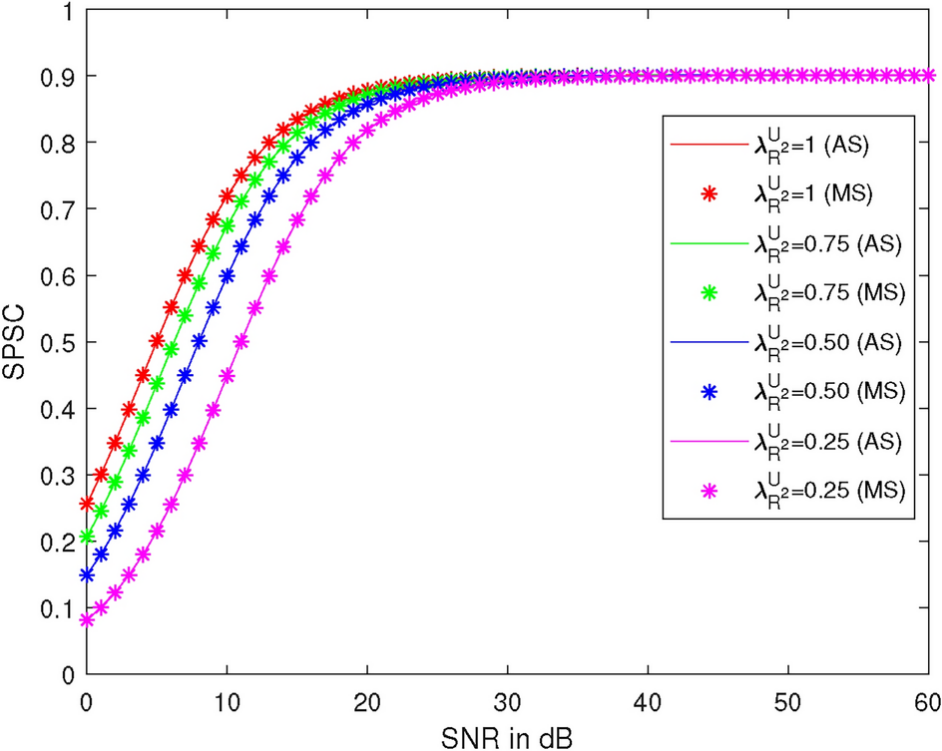
SPSC vs SNR at different values of $\lambda _R^{U_2}$.

Figure 10 illustrates the plots of SPSC versus SNR at different values of $\lambda _R^{U_2}$, specifically $0.25, 0.50, 0.75, \text {and } 1$. Here, we take $R_1 = 0.51$ BPCU, $R_2 = 2.1$ BPCU, $\lambda _{R} ^{U_1} = 1$, $\lambda _X^R = 1$, $\lambda _R^{D_2} = 1$, the power of the first allocation factor = 0.87, and $\rho _2 = 1$ dB. At 10 dB SNR, the SPSC values are 0.719, 0.674, 0.599, and 0.448 at $\lambda _R^{U_2} = 1, 0.75, 0.50, \text {and } 0.25$, respectively. SPSC decreases by 6% at $\lambda _R^{U_2} = 0.75$, 17% at $\lambda _R^{U_2} = 0.50$, and 38% at $\lambda _R^{U_2} = 0.25$ compared to $\lambda _R^{U_2} = 1$. It is observed that SPSC increases as $\lambda _R^{U_2}$ decreases and also with $\rho _1$ up to 30 dB.

## Conclusion

This paper proposed an AF relay based UAV assisted cooperative NOMA system with two untrusted destinations. The exact expression of SOP and SPSC were derived. Simulation results shows that SOP increases with higher values of average illegal SNR, higher target rate and lower values of power allocation coefficient of far destination. Also, SPSC increases with lower values of average illegal SNR, higher values of power allocation coefficient of far destination, higher values of Rayleigh channel parameter between relay and untrusted far destination and untrusted near destination. Furthermore, with high values of average legal SNR, there is a better chance of obtaining a secure communication which leads to the conclusion that in a UAV assisted cooperative NOMA system, the secrecy performance is usually determined by power allocation coefficient, average illegal SNR, target rate and Rayleigh channel parameter between relay and untrusted destinations. Therefore, it is observed from the simulation results that secrecy performance stabilizes at high legal SNR values, showing that enhanced security is not always achieved by only increasing transmission power. Rather, it may be more efficient to optimize system design at lower and moderate SNR values. This analysis may be helpful for providing privacy, integrity and reliability to wireless destinations in presence of untrusted destinations who have strong eavesdropping capabilities. In the future, the full duplex relay and multiple untrusted destinations may be used to analyze the proposed system model. Additionally, future work will include integrating intelligent power allocation through deep learning, adaptive beamforming for UAV mobility, or cooperative jamming strategies to enhance secrecy performance in UAV-assisted NOMA networks.

## Data Availability

The data used and/or analyzed during the current study are available from the corresponding author upon reasonable request.
